# Validation of Persian Individualized Neuromuscular Quality of Life in patients with muscular dystrophies

**Published:** 2020-01-05

**Authors:** Kamyar Moradi, Shirin Jamal-Omidi, Maryam Masoudi, Sayna Bagheri, Shahriar Nafissi, Farzad Fatehi

**Affiliations:** 1 Department of Neurology, School of Medicine, Tehran University of Medical Sciences, Tehran, Iran; 2 Iranian Center of Neurological Research, Neuroscience Institute, Tehran University of Medical Sciences, Tehran, Iran; 3 Shariati Hospital, Tehran University of Medical Sciences, Tehran, Iran

**Keywords:** Quality of Life, Neuromuscular Diseases, Surveys and Questionnaires, Validation Study, Persian

## Abstract

**Background:** Neuromuscular disorders affect physical and mental aspects of a patient and in other words alter the patients’ quality of life (QOL). In the present study, we investigated the validity and reliability of the Persian version of Individualized Neuromuscular QOL (INQOL) to provide a better insight into patients’ QOL.

**Methods:** Original version of the INQOL was translated backward and then forward. The resultant Persian version and a standard questionnaire, 36-Item Short Form Health Survey (SF-36), were then given to 83 participants with neuromuscular disorders. Internal consistency, known-group validity, concurrent validity, and test-retest reliability were assessed.

**Results:** The scores of matched sections for QOL in the two questionnaires were favorably correlated (P < 0.05). Correlation between test and retest scores was also significant (P < 0.05). Moreover, the Cronbach’s alpha of 0.82 was representative of robust internal consistency between INQOL covering sections.

**Conclusion:** The Persian version of the INQOL can be used in clinical and research practice to detect changes in QOL which are related to neuromuscular disorders, due to its favorably reliable and valid characteristics.

## Introduction

Muscular dystrophies are a group of degenerative muscle diseases characterized by progressive muscle wasting and weakness.^1^ They can cause a wide variety of symptoms usually affecting different aspects of patients' life. Although a curative treatment for muscular dystrophies does not yet exist, assessing patients’ quality of life (QOL) can bring about better understanding, thus guiding palliative therapies to improve various aspects of their lifestyle despite the disease progression.^2^

In several previous studies, generic health-related QOL (HRQOL) questionnaires such as 36-Item Short Form Health Survey (SF-36) have been used to quantify the QOL in patients with muscle disorders.^3^^-^^5^ As these questionnaires are not particularly designed for muscle disorders, they provide a limited insight into QOL in this group of patients. To attain a better picture on QOL, relatively specific tools could have been employed to cover more features related to muscle disorders. Regarding the issue, Individualized Neuromuscular QOL (INQOL) questionnaire was formulated and validated using qualitative interviews and a postal survey.^6^ INQOL consists of 45 questions within 10 sections, with each demonstrating one aspect of QOL in patients suffering from neuromuscular disorders and overall attempting to take a more comprehensive look. Specific aspects include the severity of muscle symptoms, the impact of muscle symptoms on particular features of life, and finally the response to treatment.^6^ The validity, reliability, and responsiveness of INQOL collectively make it an acceptable alternative to generic HRQOL questionnaires in this specific group.^2^

Despite INQOL apparent usefulness and relevance for research and clinical settings, it has only been validated in few countries [i.e., in the United Kingdom (UK),^6^ Italy,^7^ the United States of America (USA),^8^ Netherlands,^9^ Spain,^10^ and Serbia^11^]. In Iran, however, the QOL for patients with muscle abnormalities has been currently assessed by the SF-36 questionnaire. To address this issue, we translated the INQOL to Persian language and designed this study to evaluate its validity and reliability.

## Materials and Methods

***INQOL:*** INQOL contains 45 questions within 10 sections. The first four parts determine the severity of common muscle disease symptoms (weakness, fatigue, locking, and pain). Next 5 sections highlight the disease impact on different areas of life (activities, independence, social relationship, emotion, and body image), and the last item measures the treatment status and the subject’s opinion. Items are evaluated by a 7-point Likert scale assessing the degree of impact of muscle disorders on the score of each domain. The score of each of the first 9 items is calculated in percentage, and the overall ratings of sections concerning the impact on particular life aspects are reported as the QOL Index. The last item score is divided into two subscores measuring the treatment convenience and the patient expectation of treatment, respectively. To summerise, INQOL assesses ten life aspects influenced by muscle diseases, presented as 11 subscores and a total score.^6^

***Recruitment and data collection*** *:* Our participants were all cases of muscular dystrophy, recruited from the outpatient muscle clinics of Shariati Hospital, Tehran, Iran, or the private office of a neuromuscular specialist from August 6, 2017 to March 1, 2018. The inclusion criteria for taking part in this survey were the confirmed diagnosis of muscular dystrophy by the standard clinical and paraclinical criteria for the diagnosis including progressive muscle wasting and weakness, abnormal muscle biopsy, elevated level of creatine phosphokinase (CPK), genetic tests, or electromyography (EMG)-nerve conduction velocity (NCV). Moreover, the subjects had to be 12 years or older and literate in Persian, and their disease had to be symptomatic at least for a month. Participants suffering from cardiac or respiratory conditions unrelated to their diseases were excluded from our study. A total of 83 patients with different muscle diseases were eligible for enrolling. Before entering the study, the purpose of the survey was explained to all the patients and the written informed consent was taken. This study was approved by Institutional Review Board (IRB) of Tehran University of Medical Sciences, Tehran, and conducted in accordance with the Declaration of Helsinki.

***Translation and adaptation:*** Before the start, the official permission from the original developer team of INQOL was obtained. Afterward, four individuals (including a specialist in neuromuscular diseases) translated the original version of the INQOL questionnaire into Persian. The translations were debated by the executive team and the reconciled form was used as our first draft. Later, the accepted drafts were translated back to English by an expert Persian-English translator to be assured of the conceptual equivalence.

***Design:*** Our study was conducted in two main phases. In the first step, all of the recruited patients filled the INQOL questionnaire as well as the validated Persion version of SF-36, which is one of the most widely-used QOL questionnaires in muscular disorders.^12^ Consequently, their severity condition was measured by Muscular Impairment Rating Scale (MIRS) in myotonic patients^13^ and by modified Walton & Gardner-Medwin (WGM) scale^14^ in others. Additional data regarding their age, gender, disease duration, CPK levels, comorbidities, and family history were gathered for most participants in the outpatient setting. One month later in the second phase, 36 randomly-chosen patients refilled INQOL for test-retest reliability assessment. One month was short enough not to change their life quality but at the same time long enough to discard the recall effect.

We subdivided our patients into two groups of myotonic and non-myotonic as their severity evaluation method was different. For the reliability, we assessed the internal consistency of the questionnaire with Cronbach’s alpha; also we tested the reliability with performing the test-retest intraclass correlation coefficient (ICC) and calculating Spearman’s correlation. For the validity, the questionnaire validity was examined in two aspects of the known-group and concurrent validity. The known-group validity compares the obtained score among the distinct class of subjects expected to differ on the desired variable, namely the severity. In this study, we examined the between-group difference regarding the severity, gender, comorbidity presence, CPK level, and age. The concurrent validity was assessed by determining the correlation between the corresponding subscales of the INQOL and SF-36 extracted from the original article of developing and validating INQOL (the INQOL pain section vs. SF-36 bodily pain section, the INQOL activities vs. SF-36 physical functioning, the INQOL emotions vs. SF-36 emotional well-being, and the INQOL social relationship vs. SF-36 social function).

For analyzing our sample, we used SPSS software (version 24, IBM Corporation, Armonk, NY, USA). As our respondent population did not satisfy the assumption of normality, we used nonparametric tests (Mann-Whitney U test or the Kruskal-Wallis test), and for computing the between-group difference and for all correlations, we used Spearman's rho. We considered the correlation above 0.40 with a moderate relationship to be acceptable and above 0.60 with a strong concordance. Concerning the ICC, we used a mixed effect model with the acceptance level of 0.70.

## Results

A total of 83 eligible subjects entered the study, suffering from different muscular dystrophies which the majority of them had limb-girdle muscular dystrophy (LGMD) and myotonic dystrophy. The samples characteristics are summarised in table 1. Comparing myotonic and non-myotonic subgroups, we found that all items except for pain and treatment differed significantly (Table 2). 

**Table 1:** Participants’ characteristics

Disease spectrum	LGMD: 50
	Myotonic dystrophy: 16
	FSHD: 10
	BMD: 5
	DMD: 1
	EDMD: 1
Age (year)	12-72 (median: 31.5)
Sex (male/female)	49/34
Duration (year)	1-30 (median: 9.8)
CPK	10: normal CPK
	26: 2-10 fold increase in CPK
	38: > 10 fold rise
	9: CPK has not been checked
Family history	No positive FH: 28
	First-degree FH: 38
	Second-degree FH: 17
Severity	16 myotonic patients, MIRS score: 2.8 ± 0.9
	67 non-myotonic patients, modified WGM score: 4.2 ± 2.1
Respiratory complication of the disease	Examined with spirometry in 30 patients: present in 6 of them
Cardiac complication of the disease	Examined with echocardiography in 72 patients: present in 4 of them

LGMD: Limb-girdle muscular dystrophy; FSHD: Facioscapulohumeral muscular dystrophy; BMD: Becker muscular dystrophy; DMD: Duchenne muscular dystrophy; EDMD: Emery-Dreifuss muscular dystrophy; CPK: Creatine phosphokinase; MIRS: Muscular Impairment Rating Scale; WGM: Walton & Gardner-Medwin scale; FH: Familial hypercholesterolemia

**Table 2:** Comparison of Individualized Neuromuscular Quality of Life (INQOL) subsections between myotonic and non-myotonic dystrophies

**INQOL items**	**Diagnosis**	**Mean ± SD**	**Mann-Whitney U**	**P**
Weakness	Non-myotonic	69.2 **± **19.1	195.5	< 0.01
	Myotonic	37.2 ± 28.1		
Locking	Non-myotonic	3.9 ± 14.4	125.0	< 0.01
	Myotonic	30.3 ± 24.0		
Pain	Non-myotonic	32.3 **±** 28.7	396.0	0.10
	Myotonic	19.1 **±** 20.7		
Fatigue	Non-myotonic	59.2 ± 24.6	273.0	< 0.01
	Myotonic	34.9 ± 26.8		
Activities	Non-myotonic	59.5 ± 23.6	185.5	< 0.01
	Myotonic	30.6 ± 18.9		
Independence	Non-myotonic	49.1 ± 30.6	250.5	< 0.01
	Myotonic	20.0 ± 21.8		
Social relationship	Non-myotonic	34.3 ± 24.5	205.5	< 0.01
	Myotonic	9.0 ± 15.2		
Emotions	Non-myotonic	42.9 ± 27.4	272.5	< 0.01
	Myotonic	19.3 ± 13.2		
Body image	Non-myotonic	51.4 ± 29.1	275.5	< 0.01
	Myotonic	23.8 ± 30.2		
QOL	Non-myotonic	51.5 ± 21.5	197.0	< 0.01
	Myotonic	26.3 ± 18.1		
Perceived treatment	Non-myotonic	14.7 ± 21.2	473.0	0.46
	Myotonic	20.3 ± 32.3		
Expected treatment	Non-myotonic	18.2 ± 22.6	485.0	0.55
	Myotonic	21.9 ± 31.6		

INQOL: Individualized Neuromuscular Quality of Life; QOL: Quality of life; SD: Standard deviation

***Reliability:*** By evaluating the internal consistency, the overall scale Cronbach’s alpha for baseline data was 0.82 with the item-total values above the acceptable limit of 0.70 ranging from 0.78 for QOL Index to 0.85 for locking section (Table 3).^15^ As we omitted the treatment items from the scale, regarding the previous INQOL validation projects,^16^ our internal consistency significantly improved (the overall estimation: 0.85). Moreover, the measured Cronbach's alphas in the myotonic and non-myotonic group were 0.79 and 0.81, respectively. 

Comparing the ICC and the correlation between 36 subjects’ test-retest scores revealed that ICCs for all the items were above 0.90 except for the treatments and emotion items which were above 0.80, satisfying the repeatability condition. Also, the Cronbach’s alpha in the retest group was assessed (0.80) with all of the item-deleted values above 0.70 (Table 3).

**Table 3:** Test-retest reliability

**INQOL items**	**ICC**	**CI (95%)**	**Correlation ** **coefficient**	**P**	**Cronbach’s alpha ** **(test)**	**Cronbach’s alpha ** **(retest)**
Weakness	0.93	0.88-0.97	0.87	< 0.01	0.78	0.76
Locking	0.94	0.90-0.97	0.99	< 0.01	0.85	0.82
Pain	0.91	0.84-0.95	0.88	< 0.01	0.81	0.77
Fatigue	0.93	0.88-0.97	0.93	< 0.01	0.79	0.76
Activities	0.93	0.87-0.97	0.93	< 0.01	0.78	0.76
Independence	0.92	0.85-0.96	0.91	< 0.01	0.79	0.77
Social relationship	0.97	0.96-0.99	0.98	< 0.01	0.79	0.78
Emotions	0.83	0.69-0.91	0.80	< 0.01	0.80	0.75
Body image	0.95	0.90-0.97	0.95	< 0.01	0.80	0.77
QOL	0.93	0.87-0.96	0.92	< 0.01	0.78	0.75
Perceived treatment	0.80	0.64-0.89	0.78	< 0.01	0.84	0.84
Expected treatment	0.88	0.78-0.94	0.89	< 0.01	0.84	0.83

ICC: Interclass correlation coefficient; CI: Confidence interval; INQOL: Individualized Neuromuscular Quality of Life; QOL: Quality of life

**Table 4:** Association between Individualized Neuromuscular Quality of Life (INQOL) components and muscular disorders severity

**INQOL items**	**Myotonic patients**		**Non-myotonic patients**	
	**P-value for severity**	**P-value for CPK level**	**P-value for severity**	**P-value for CPK level**
Weakness	0.02	0.38	0.02	0.18
Locking	0.29	0.19	0.17	0.54
Pain	0.25	0.86	0.82	0.47
Fatigue	0.11	> 0.99	0.26	0.83
Activities	0.17	0.10	0.11	0.24
Independence	0.15	0.10	< 0.01	0.19
Social relationship	0.29	0.38	0.83	0.26
Emotions	0.34	0.38	0.32	0.57
Body image	0.36	0.86	0.52	0.03
QOL	0.17	0.38	0.95	0.15
Perceived treatment	0.59	0.10	0.38	0.33
Expected treatment	0.81	0.10	0.77	0.58

INQOL: Individualized Neuromuscular Quality of Life; CPK: Creatine phosphokinase; QOL: Quality of life

***Known-group validity:*** We evaluated the validity in two dimensions: known-group (psychometric) and concurrent. We compared the score of items in groups based on the severity, gender, presence of comorbidity, CPK level, and age. Regarding the severity, in the non-myotonic group, the mean score differed significantly in the weakness and independence sections (P = 0.02 and P < 0.01, respectively); while in the myotonic group, it was only valid for the weakness section (P = 0.02) (Table 4). 

Also, we could not find any significant association between the presence of comorbidity, age, gender and questionnaire scores. Moreover, we could not find any correlation between CPK levels and items of the questionnaires except for body image section in non-myotonic patients (Tables 5 and 6).

***Concurrent validity:*** We assessed the correlation between the analogous sections of SF-36 and INQOL. All the corresponding sections were highly associated in two questionnaires with the most substantial concordance presented for emotion section (Table 7). As well, the overall estimation of QOL proposed as QOL Index on INQOL and the SF-36 general health index were moderately related (Spearman's rho: 0.57).^17^

## Discussion

In this study, we translated and adapted the original form of INQOL to Persian language and evaluated its reliability and validity in the target population. The results indicated that the Persian version could be reliably and validly applied to measure the QOL in patients with muscular dystrophies. Comparison between INQOL parameters and corresponding sections of SF-36 suggested that INQOL could be alternatively utilized to estimate QOL for neuromuscular patients.

**Table 5:** Association between gender and Individualized Neuromuscular Quality of Life (INQOL) items

**INQOL items**	**Gender**	**Mean ± SD**	**P**
Weakness	Male	62.74 ± 25.84	0.93
	Female	63.47 ± 22.80	
Locking (myotonia)	Male	9.56 ± 20.40	0.76
	Female	8.24 ± 18.54	
Pain	Male	29.50 ± 27.56	0.93
	Female	30.16 ± 28.40	
Fatigue	Male	55.83 ± 24.22	0.87
	Female	52.61 ± 30.13	
Activities	Male	57.38 ± 25.13	0.13
	Female	49.04 ± 25.34	
Independence	Male	48.96 ± 31.12	0.06
	Female	35.57 ± 30.09	
Social relationship	Male	30.80 ± 25.86	0.70
	Female	27.42 ± 24.09	
Emotions	Male	41.76 ± 26.01	0.15
	Female	33.41 ± 27.74	
Body image	Male	47.55 ± 30.24	0.58
	Female	43.90 ± 32.57	
QOL	Male	50.79 ± 22.92	0.06
	Female	40.70 ± 22.31	
Perceived treatment	Male	14.45 ± 24.75	0.33
	Female	17.64 ± 22.17	
Expected treatment	Male	20.75 ± 24.83	0.26
	Female	16.17 ± 23.92	

INQOL: Individualized Neuromuscular Quality of Life; QOL: Quality of life; SD: Standard deviation

The results of SF-36 and INQOL demonstrated an inverse correlation with each other due to the different direction of the two scoring systems. Moreover, in line with the studies validating the INQOL in Italy^7^ and Netherlands,^9^ we observed a steady association between INQOL and SF-36 questionnaires; however, the strongest correlation was observed in the emotion section which is in total discordance with the findings of the studies mentioned above.

**Table 6:** The association of age and comorbidity with Individualized Neuromuscular Quality of Life (INQOL) items

**INQOL items**	**P-value ** **for age**	**P-value for presence ** **of comorbidity**
Weakness	0.39	0.42
Locking	0.92	0.50
Pain	0.68	0.87
Fatigue	0.47	0.83
Activities	0.60	0.55
Independence	0.17	0.95
Social relationship	0.47	0.85
Emotions	0.99	0.31
Body image	0.40	0.59
QOL	0.47	0.93
Perceived treatment	0.62	0.29
Expected treatment	0.30	0.31

INQOL: Individualized Neuromuscular Quality of Life; QOL: Quality of life

According to the study of Sansone et al.,^7^ physical concepts more strongly correlated with SF-36 items compared to mental aspects; nevertheless, our investigation demonstrated a considerable correlation between mental sections of INQOL and SF-36 items, as well. 

**Table 7:** Summary of concurrent validity

**Comparison ** **(Spearman’s rho) **	**Correlation ** **coefficient**	**P**
Pain SF-36 vs. pain INQOL	-0.88	< 0.01
Emotion SF-36 vs. emotion INQOL	-0.91	< 0.01
Physical activity SF-36 vs. activities INQOL	-0.63	< 0.01
Social function SF-36 vs. social relationship INQOL	-0.80	< 0.01
General health SF-36 vs. QOL index INQOL	-0.57	< 0.01

INQOL: Individualized Neuromuscular Quality of Life; QOL: Quality of life; SF-36: 36-Item Short Form Health Survey

Our test-retest analysis supported the excellent reliability of INQOL questionnaire which is consistent with the previous reports.^7^^,^^11^ Moreover, several domains including locking, social relationships, and body image exhibited the least amount of alteration, proposing them as the most stable life aspects in a patient with a muscle disorder.

In both myotonic and non-myotonic dystrophic patients, severity of the disease showed significant correlation with weakness as a physical index, but not with mental components of INQOL. This is relatively in accordance with the physical-based construction of INQOL. The weakness section of INQOL inversely correlated with severity of myotonic dystrophies, as expected. However, surprisingly, the rest scores of INQOL did not followed a similar trend. On the other hand, INQOL known-group validation in Serbia resulted in mostly higher significant scores of the items proportionate with the severity.^11^ This finding indicates that with entering a larger myotonic population, INQOL could have been validated more efficiently. On the other hand, independence section was implicated beside the weakness item in non-myotonic dystrophic patients. Muscular dystrophies could involve multiple systems including muscle strength, cognition, respiratory function, and gastrointestinal (GI) system and patients’ mobility and self-care tasks might be impaired in these disorders due to entanglement of sundry systems.^11^^,^^18^ Hence, with the progression of disease, patients could feel more dependent as they would become too incapacitate to organize their self-care tasks precisely. 

Our results showed that the treatment-related scores correlated with none of the SF-36 sections. It emphasizes the fact that muscular dystrophies do not take significant advantages from conventional treatments. Medication therapies, physiotherapy, and ergotherapy can only decelerate the progression of muscular disorders.^19^ In myotonic dystrophy also, the symptomatic therapies are ineffective on the progression of muscle wasting and strength improvement. As these treatments could not considerably improve patients’ condition, sections related to perceived and expected treatments did not appear to be associated with QOL of the patients. Furthermore, both questionnaires express patient’s status in the present time while the treatment-related questions ask patients to compare their present condition with prior to the beginning of treatment and with an image of themselves in the future that they would expect to achieve. 

The myotonic section of INQOL was correlated with none of SF-36 sections which was in line with a few of previous corresponding studies.^7^^,^^11^ As mentioned, SF-36 questionnaire has not been applied exclusively for a patient with muscular dystrophy, since none of the items examines myotonic symptoms. The most common symptom observed in patients with myotonic dystrophy is the locking of distal muscles frequently progressing to proximal parts through the passage of time;^13^ as a result, SF-36 that mostly concentrates on weakness of proximal muscles cannot be considered as a valid tool for the QOL assessment in a patient with myotonic dystrophy especially for type 1. However, the number of patients with myotonic dystrophy who entered our study was low; therefore, our insufficient results of this section could not be generalized.

Our study had several limitations: First, although all enrolled subjects suffered from similar symptoms such as progressive muscle weakness, fatigue, and pain, the study population was still heterogeneous due to recruitment of patients with different muscular dystrophies. The major problem with evaluation of QOL in each specific muscle disorder is the rarity of patients and therefore, small sample size. However, further investigations are highly recommended to examine QOL in muscular dystrophies specifically. Second, the source of INQOL construction is the International Classification of Impairments, Disabilities, and Handicaps (ICIDH-2) model of disease, which mostly concentrates on the physical disability rather than QOL.^6^ Third, our study was cross-sectional in design and hence, responsiveness of the Persian version of INQOL was not measured. This limitation was due to the progressively debilitating nature of muscle disorders leading to patients’ little cooperation. However, regarding the slow aggravating essence of muscular disorders,^20^ we highly suggest further investigations following the patients at least for five years. Finally, a larger sample size following normal distribution could help us report the validity and reliability of the Persian version more confidently.

## Conclusion

Exploring the acceptance of INQOL questionnaire for determining QOL in the Persian patients with muscular dystrophies resulted in substantial reliability, internal consistency, and concurrent validity. This report suggests INQOL as a valid and reliable tool for evaluation of QOL in neuromuscular patients in further clinical and research fields.

## Notes:

**How to cite this article:** Moradi K, Jamal-Omidi S, Masoudi M, Bagheri S, Nafissi S, Fatehi F. Validation of Persian Individualized Neuromuscular Quality of Life in patients with muscular dystrophies. Curr J Neurol 2020; 19(1): 13-9.
